# Managing Major Postpartum Haemorrhage following Acute Uterine Inversion with Rusch Balloon Catheter

**DOI:** 10.1155/2011/541479

**Published:** 2011-06-26

**Authors:** Remon Keriakos, Smriti Ray Chaudhuri

**Affiliations:** ^1^Department of Obstetric and Gynaecology, Jessop Wing, Royal Hallamshire Hospital, Sheffield Teaching Hospitals NHS Foundation Trust, Tree Root Walk, Sheffield S10 2SF, UK; ^2^Sheffield Teaching Hospitals NHS Foundation Trust, Sheffield S10 2SF, UK

## Abstract

Acute postpartum uterine inversion is a relatively rare complication. The uterus inverts and the uterine fundus prolapses to or through the dilated cervix. It is associated with major postpartum haemorrhage with or without shock. Shock is sometimes out of proportion to the haemorrhage. Minimal maternal morbidity and mortality can be achieved when uterine inversion is promptly and aggressively managed. We present this report of three cases of acute uterine inversion complicated with major postpartum haemorrhage and managed with Rusch balloon. The paper highlights the importance of early recognition and the safety of the use of intrauterine balloon to manage major postpartum haemorrhage in these cases.

## 1. Introduction

Acute postpartum uterine inversion is a relatively rare complication. The uterus inverts and the uterine fundus prolapses to or through the dilated cervix. It is associated with major postpartum haemorrhage with or without shock. Shock is sometimes out of proportion to the haemorrhage. Minimal maternal morbidity and mortality can be achieved when uterine inversion is promptly and aggressively managed. We present this report of three cases of acute uterine inversion complicated with major postpartum haemorrhage and managed with Rusch balloon. The case series highlights the importance of early recognition and the safety of the use of intrauterine balloon to manage major postpartum haemorrhage in these cases.

## 2. Case Report

*The first case* was a 28-year-old lady in her first pregnancy who was admitted in spontaneous labour at 41 weeks of gestation. There were no known antenatal risk factors for PPH. She had spontaneous vaginal delivery of a female baby weighing 3.5 kg at the hospital midwifery unit. The first stage of labour lasted for eight hours. Intramuscular syntocinon was given after delivery of the baby. The placenta was delivered 20 minute after delivery of the baby by her midwife by controlled cord traction after two tractions. She continued to bleed following the delivery of the placenta and hence the obstetric registrar was called. She was found to have acute postpartum uterine inversion. The diagnosis was made 10 minutes following the delivery of the placenta. The initial recorded blood loss was 1000 mL when uterine inversion was diagnosed. She was taken to theatre and a manual replacement was done for correction of inversion. Medical management included syntocinon infusion, syntometrine, and 2 doses of hemabate (carboprost). To prevent the recurrence of the inversion and manage the persistent haemorrhage, a Rusch balloon catheter was inserted. The insertion was initially performed by senior registrar with 700 mL of saline but the balloon prolapsed through the cervix. The Rusch balloon was reinserted by consultant with 720 mL of saline. It was successful in arresting the blood loss and the total estimated loss was 4000 mL. A vaginal pack was inserted and IV antibiotics were prescribed for 24 hr as per protocol. She received four units of blood transfusion and three units of fresh frozen plasma. The Rusch balloon was planned for removal in 12 hours but it burst spontaneously in approximately 10 hours time. There was no further blood loss and no postnatal complications were recorded.

*The second case* was a 25-year-old lady in her first pregnancy who was admitted at 39+4 weeks of gestation. She had a BMI of 30 and underwent induction of labour with propess for reduced fetal movement and persistent proteinuria. She then had artificial rupture of the membranes and syntocinon infusion. She had a spontaneous vaginal delivery and gave birth to a female baby weighing 3.3 kg. The placenta was delivered by controlled cord traction. The first stage lasted for six hours. The placenta was delivered 15 minutes after delivery of the baby. She continued to bleed following the delivery of the placenta and the registrar was called. An acute postpartum uterine inversion was diagnosed 5 minutes following the delivery of the placenta. Manual replacement was performed in theatre to correct the inversion. She was continued on syntocinon infusion and was also given intravenous ergometrine, 2 doses of intramuscular and intramyometrial hemabate (carboprost), and Misoprostol 800 micrograms rectally. There was persistent uterine atony and haemorrhage. Estimated blood loss was 4 liters. She was given 5 units of blood, 2 unit fresh frozen plasma and cryoprecipitate. Rusch balloon catheter was inserted to control the haemorrhage. The procedure was performed by senior registrar and 650 mL of saline was used for inflation of the balloon. A vaginal pack was inserted and IV antibiotics were prescribed for 24 hours and oral for further 5 days. Rusch balloon was successful and removed after 24 hours. Removal of catheter was easy and there were no complications recorded.

*The third case* was a 20-year-old lady in her second pregnancy who was admitted in spontaneous labour at 36 + 3 weeks of gestation. There were no known antenatal risk factors. She had a ventouse delivery for prolonged second stage of labour. The first stage lasted for seven hours. Intramuscular syntometrine was given. It was documented that the placenta and membranes were expelled spontaneously by maternal effort. She had postpartum haemorrhage following the delivery of the placenta and acute uterine inversion was diagnosed 5 minutes after delivery of the placenta. The patient was immediately transferred to theatre and manual replacement of uterus was performed. She was given syntocinon infusion, syntometrine intramuscular, and one dose of hemabate. She had severe postpartum haemorrhage due to uterine atony. The estimated blood loss was 2.5 liters. The consultant on call inserted Rusch balloon catheter which was inflated with 700 mL normal saline. Vaginal pack was inserted and intravenous antibiotics were prescribed for first 24 hrs as per protocol. She had 4 unit of blood transfusion. Management with Rusch balloon was successful, and it was removed after 12 hours of insertion. Removal of the catheter was easy and no further postpartum problems were recorded.

## 3. Discussion

Acute uterine inversion is a rare obstetric emergency. In 30, 466 deliveries over 4.5 years we had three cases giving an incidence of about 1 : 10.000. All of them were recognized early but had major postpartum haemorrhage. There have been only very few reported cases of managing acute uterine inversion and related major postpartum haemorrhage with hydrostatic balloon [1, 2]. Although acute uterine inversion is relatively uncommon, it remains a potentially life threatening obstetric emergency. The potential for maternal morbidity is significant high if it is not recognised and corrected quickly by repositioning of the uterine fundus. Maternal mortality can be as high as 15% [3].

Postpartum acute uterine inversion is an obstetric emergency where the uterine fundus passes through the cervix (complete inversion), or remains above this level (incomplete inversion).

Presentation of uterine inversion can be acute (within 24 hours of delivery), subacute (over 24 hours and up to the 30th postpartum day) or chronic (more than 30 days after delivery) [4].

Various aetiological factors have been linked to uterine inversion, though in the majority of cases no obvious causes are found. Attributable factors include short umbilical cord, excessive traction on the umbilical cord, fundal pressure, fundal implantation of the placenta, abnormal adherence of the placenta, rapid or long labours, previous uterine inversion, and certain drugs such as magnesium sulphate and other tocolytic drugs.

It presents most often with symptoms of a postpartum haemorrhage. In a series of 40 cases, postpartum haemorrhage complicated 65% of cases of acute uterine inversion, and 47.5% required blood transfusion [5]. There was no recurrence in 26 subsequent deliveries [5]. Early recognition and intervention could reduce the risk of complications from major postpartum haemorrhage including disseminated intravascular coagulopathy and other related morbidity. All our cases had major postpartum haemorrhage and required blood transfusion but recognized early and managed with blood transfusion and intrauterine Rusch balloon insertion. The classic presentation of acute inversion as in our cases is postpartum haemorrhage, or/and sudden appearance of a vaginal mass and/or postpartum collapse which in some cases of complete uterine inversion is out of proportion to blood loss. Reason for initial shock is generally thought to be neurogenic, due to the traction effect on the surrounding peritoneum which is classically associated with bradycardia [6]. Pain can be severe. The diagnosis is usually suspected when it is difficult to feel the uterine fundus. Diagnosing a first-degree inversion is much more difficult. Obesity can make diagnosis more difficult. Chronic cases are unusual and difficult to diagnose. They may present with spotting, discharge, and low-back pain. Ultrasound may be required to confirm the diagnosis. 

Acute uterine inversion can be life threatening; however, it can be successfully managed with rapid recognition, intravenous fluids, blood transfusion, and immediate repositioning of the uterus and medical management for atonic uterus. The use of intrauterine balloon has proven to be useful to reduce blood loss and prevent recurrence as in our cases.

Prompt replacement of the uterus is best done manually and promptly as delay can render replacement progressively more difficult. Placenta should be left if still attached.

Johnson's technique refers to the manual replacement method where the uterus is replaced by placing a fist on the fundus and gradually pushing it back into the pelvis through the dilated cervix. Bimanual uterine compression and massage should be maintained until the uterus is well contracted and bleeding has stopped. Should manual reduction fail to achieve uterine repositioning, then employing the use of hydrostatic replacement with Rusch balloon [1] or SOS Bakri Balloon [2] or O'Sullivan's technique is usually the next approach. The urological Rusch balloon has been described as preferable method in replacement of inverted uterus and managing major postpartum haemorrhage by virtue of larger capacity, ease of use, and low cost [1, 7, 8]. Tocolytics like terbutaline, nitroglycerine, or magnesium sulphate can be used to facilitate repositioning.

If the above are unsuccessful a surgical approach is required. Huntington procedure involves a laparotomy to locate the cup of the uterus. Allis forceps is used to gently apply upward traction until the inversion is corrected [9]. Haultain technique involves a longitudinal incision on the posterior cervical ring and reversal by gentle traction as in the Huntington procedure [10]. Antonelli et al. 2006 [11] has advocated using a silastic cup to facilitate reduction of uterine inversion by gentle upward pressure, performed at the time of laparotomy. Laparoscopic technique to reduce uterine inversion has been recently described as well [12]. Hysterectomy is the last resort when other surgical methods are unsuccessful.

## 4. Conclusion

Uterine inversion is rare but carries high morbidity including major postpartum haemorrhage and blood transfusion. Delay in the diagnosis can lead to serious complications related to major postpartum haemorrhage. Increasing awareness regarding the detection and a multidisciplinary approach are essential components of management. Urgent replacement of the uterus, resorting early to blood transfusion, and use of uterine balloon catheter would reduce the morbidity from this condition. The advantage of Rusch balloon over other balloons is the capacity which no other balloon has. Though the use of Rusch balloon in major postpartum haemorrhage is not a new technique, this case reports highlight the safety of using it in cases of acute inversion complicated with postpartum haemorrhage and also add to the few reported cases on its safety in managing the acute inversion, related major postpartum haemorrhage, and possibly prevention of recurrence.

##  Conflict of Interests

The authors declare that there is no conflict of interests.
